# Clinical and economic comparison of an individualised immunoglobulin protocol vs. standard dosing for chronic inflammatory demyelinating polyneuropathy

**DOI:** 10.1007/s00415-018-9157-4

**Published:** 2018-12-17

**Authors:** Yusuf A. Rajabally, Saadia Afzal

**Affiliations:** 1 Regional Neuromuscular Service, University Hospitals Birmingham, Birmingham, UK; 2 School of Life and Health Sciences, Aston Brain Centre, Aston University, Aston Triangle, Birmingham, B4 7ET UK

**Keywords:** Chronic inflammatory demyelinating polyneuropathy, Economic, Intravenous immunoglobulins, Outcome measures, Protocol

## Abstract

**Background:**

The clinical and economic implications of an individualised intravenous immunoglobulin (IVIg) protocol for chronic inflammatory demyelinating polyneuropathy (CIDP) are unknown. Comparison with standard dosing regimens has not been performed.

**Methods:**

We retrospectively studied 47 IVIg-treated subjects with CIDP over 4 years with an individualised, outcome-measured, dose-modifying protocol. We evaluated responder and remission rates, clinical improvement levels and dose requirements. We compared clinical benefits and costs with those reported with standard dosing at 1 g/kg every 3 weeks.

**Results:**

The IVIg-responder rate was 83% and the 4-year remission rate was 25.6%. Mean IVIg dose requirements were 22.06 g/week (SD:15.29) in patients on ongoing therapy. Dose range was wide (5.83–80 g/week). Mean infusion frequency was every 4.34 weeks (SD:1.70) and infusion duration of 2.79 days (SD:1.15). Mean Overall Neuropathy Limitation Scale improvement was 2.54 (SD:1.89) and mean MRC sum score improvement of 12.23 (SD:7.17) in IVIg-responders. Mean modified-INCAT (Inflammatory Neuropathy Cause and Treatment) score improvement was similar (*p* = 0.47) and mean MRC sum score improvement greater (*p* < 0.001) in our cohort, compared to the IVIg-treated arm of the ICE Study. Mean drug costs were GBP 37,660/patient/year (€ 43,309) and mean infusion-related costs of GBP 17,115/patient/year (€ 19,682), totalling GBP 54,775/patient/year (€ 62,991). Compared to standard dosing using recorded weight, mean savings were of GBP 13,506/patient/year (€ 15,532). Compared to standard dosing using dosing weight, savings were of GBP 6,506/patient/year (€ 7,482).

**Conclusion:**

Our results indicate that an individualised IVIg treatment protocol is clinically non-inferior and 10–25% more cost-effective than standard dosing regimens in CIDP.

## Introduction

Chronic inflammatory demyelinating polyneuropathy (CIDP) is the most common auto-immune neuropathy worldwide, with a prevalence of 3–5 per 100,000 in the United Kingdom [1, 2]. CIDP is treated with intravenous immunoglobulins (IVIg) as major first-line treatment.

Treatment regimens may in practice vary greatly in CIDP. Data on comparative levels of clinical benefit and costs are lacking, but are of great interest for clinical practice in view of IVIg cost and availability. Although effective non-protocolised, dose decrease to the minimal effective has long been described and advocated [3], the clinical gain and economic implications of a treatment protocol using close clinical monitoring of IVIg-treated patients to ascertain lowest possible effective dose and frequency requirements, refractoriness or remission, is not established in comparison to standard dosing at 1 g/kg every 3 weeks, as used in long-terms trials. True IVIg costs for CIDP are variable and remain unclear as well as without demonstrated clinical justification, in the absence of clinical effectiveness data from economic analyses performed to date [4, 5].

We studied a cohort of patients with CIDP, followed-up at our institution and treated with IVIg as per an outcome-measured treatment protocol, over a 4-year period. We ascertained responder and remission rates. We determined clinical improvement levels, doses used, duration and frequency of IVIg infusions. We compared clinical benefits achieved with those previously described with standard dosing regimens. We also compared costs in our cohort with the hypothetical costs using standard dosing regimens.

## Methods

We retrospectively reviewed electronic hospital records of patients with a diagnosis of CIDP attending the Inflammatory Neuropathy Clinic, at Queen Elizabeth Hospital, University Hospitals Birmingham, UK. Patients were selected on the basis of (1) meeting European Federation of Neurological Societies/Peripheral Nerve Society (EFNS/PNS) diagnostic criteria for “definite” or “probable” CIDP [6], and (2) having received IVIg treatment between July 2014 and June 2018.

IVIg-response is primarily defined in our clinical practice, by a ≥ 1-point improvement of the Overall Neuropathy Limitation Scale (ONLS) [7], excluding a grade 1 to grade 0 amelioration of the upper limb component, as not functionally meaningful. In those not meeting this requirement, patients are considered as responders if they demonstrate an improvement of ≥ 4 raw points (out of 48) of the inflammatory Rasch-built Overall Disability Scale (I-RODS) [8], and/or a ≥ 5 kg improvement of Jamar grip strength of the dominant or clinically more affected hand [9, 10]. Additionally, we also consider improvements of lesser amplitudes of the I-RODS and Jamar grip as consistent with an IVIg-response, if both are concurrently present, with, in addition, an accompanying Medical Research Council (MRC) Sum Score (MRCSS) improvement by ≥ 2 points. The MRCSS (out of 80) is determined by adding individual MRC scores from eight muscle groups bilaterally, including shoulder abductors, elbow flexors, wrist extensors, finger abductors, hip flexors, knee flexors, ankle dorsiflexors and extensor hallucis.

**Fig. 1:**
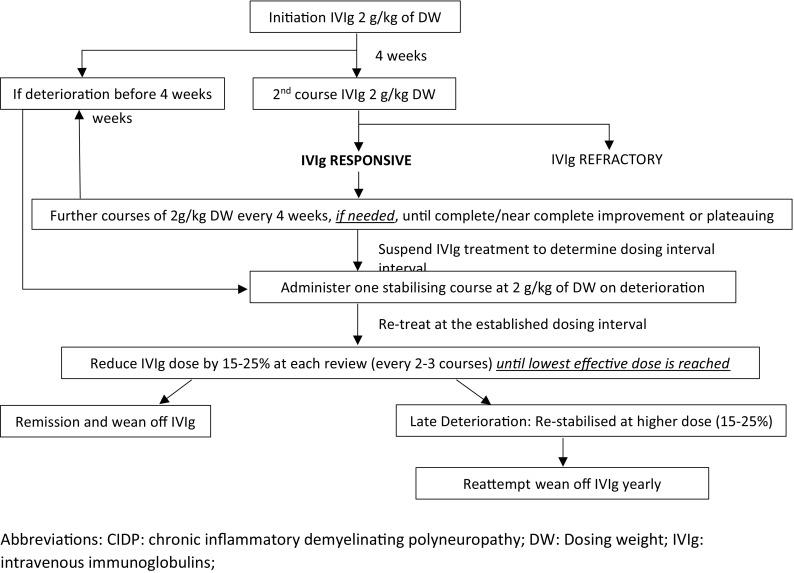
IVIg outcome-measured dose-modifying protocol for CIDP. Abbreviations: *CIDP* chronic inflammatory demyelinating polyneuropathy, *DW* dosing weight, *IVIg* intravenous immunoglobulins

IVIg dose changes are implemented using the local version of a jointly previously proposed algorithm, published by others and ourselves [11]. Our protocol (Fig. 1) involves treatment initiation at 2 g/kg, using dosing weight (DW). Two courses separated by 4 weeks are attempted to determine IVIg response or refractoriness. In responders, further courses at the same dose of 2 g/kg of DW are administered at 4-week intervals, if and as necessary, until complete or near-complete improvement or plateauing of function, defined by absence of further functional amelioration after two successive courses. Courses are administered more frequently after any of the initial courses at 2 g/kg of DW, if deterioration occurs before 4 weeks. Subsequent regular reassessments are conducted with suspension of IVIg treatment to determine dosing interval. Next, with treatment administered at the established dosing interval, and after one further stabilising course at 2 g/kg of DW, weight-independent, gradual, step-wise dose reductions of 15–25% at each review (every two to three courses), are performed until the lowest effective dose is reached, or weaning achieved. Eventual 15–25% dose re-increase is performed if and when deterioration occurs at a later stage and stabilisation attempted at that dose. Decline is defined as per above-mentioned cut-offs for ONLS, I-RODS and Jamar grip strength. If unsuccessful on the first occasion following progressive dose reductions, wean is similarly re-attempted on a yearly basis.

For each patient, we identified demographics, diagnostic criteria category fulfilment, CIDP subtype, relevant associated conditions and relevant associated treatments. In each case, we ascertained the initial IVIg dose administered as per patients’ DW, calculated as per the previously published formula adopted by the 2011 Clinical Guidelines for Immunoglobulin Use, Department of Health, UK: (DW = Ideal Body Weight + [0.4 × {recorded weight − Ideal Body Weight}, except if recorded weight ≤ Ideal Body Weight, in which case DW = recorded weight) [12]. As, in our experience, recorded weight remains frequently used instead of DW in clinical practice at many prescribing institutions in the UK and elsewhere, and as long-term IVIg trials for CIDP do not make specific mention of this issue, theoretical initial IVIg drug costs were also calculated using recorded weight at treatment initiation.

Responder rate, remission rate over the study period, IVIg doses, infusion durations and frequencies were obtained from records, as documented at last follow-up. Pre- and post-treatment disability scores were retrieved. IVIg drug costs were calculated using the mean current cost per gram (as of July 2018), of the three most used brands in our unit. Hospital infusion-related non-drug costs were calculated considering current cost of a day on our Day Case Unit, although some patients were treated in their local district hospital but monitored in our clinic. As a result, total IVIg-related costs per patient on on-going treatment, were determined. Disability score improvements were compared with those achieved in previous long-term IVIg trials for CIDP using standard dosing regimens [13, 14]. Costs were similarly compared with those of these regimens using current costs at our institution [13, 14].

Statistical analysis was performed using SPSS 25.0 software. Comparisons of proportions were done using Fisher Exact Tests, and comparison of means by *t* tests. Significance was set at *p* < 0.05.

This study was an approved and registered retrospective Clinical Audit by our institutional review body at University Hospitals Birmingham, UK (Registration number: CARMS 14164, March 2018).

## Results

Amongst subjects with a diagnosis of “definite” or “probable” CIDP as per EFNS/PNS diagnostic criteria [6] attending our Inflammatory Neuropathy Clinic between July 2014 and June 2018, 47 had been treated with IVIg. Thirty-nine subjects (83%) had responded as determined by the outcome measures as defined above.

The group of responders consisted of ten women and 29 men. Mean age was 58.36 years (SD: 15.06). Thirty-six patients (92.3%) fulfilled clinical and electrophysiological diagnostic criteria for “definite CIDP” as per the EFNS/PNS Guidelines [5]. Three (7.7%) met criteria for “probable CIDP”. The majority (32; 82.1%) had typical CIDP as defined by the EFNS/PNS Guidelines [5], 6 (15.4%) had Lewis-Sumner syndrome and one (2.6%) had pure motor CIDP. Five patients (12.8%) had associated diabetes, and 6 patients (15.4%) had an associated monoclonal gammopathy of uncertain significance (MGUS). Three of the 39 responders were on concurrent immunosuppressive treatment with methotrexate, two for concurrent rheumatological indications, and one for CIDP, initiated outside our institution. Utilised outcome measures used included MRCSS and ONLS in all 39 subjects, I-RODS in 26/39 (66.7%), and Jamar grip dynamometry in 20/39 (51.3%).

Main findings relating to mean clinical amelioration with IVIg are summarized in Table 1. Mean improvements in the 39 responders were of 12.23 (SD: 7.17) for the MRCSS and 2.54 (SD: 1.89) for the ONLS. All 39 IVIg responders improved by ≥ 2 points on the MRCSS. Thirty-six of 39 responders (92.3%) improved on the ONLS by ≥ 1 point. Nineteen of 26 (73.1%) improved by ≥ 4 points on the I-RODS. Fourteen of 20 (70%) improved by ≥ 5 kg on Jamar grip dynamometry. Thus, 38/39 (97.4%) of responders improved as per defined cut-offs on at least one scale. One of the 39 responders (2.6%), improved to levels below cut-offs for both I-RODS and Jamar grip, with additional MRCSS amelioration.

**Table 1:** Mean clinical improvement achieved on different disability outcome measures with treatment in 39 IVIg-responders with CIDP on long-term therapy over a 4-year period using an outcome-measured protocol

	MRCSS Mean (SD)	ONLS Mean (SD)
39 IVIg responders	12.23 (7.17)	2.54 (1.89)
29 long-term IVIg-dependent	12.54 (5.46)	2.32 (2.00)
10 patients in remission	10.80 (6.63)	3.20 (1.40)

Comparative statistical analysis of clinical amelioration was possible with one of the two previous trials with standard dosing [13] but not the other [14], and for the modified-INCAT (Inflammatory Neuropathy Cause and Treatment) and MRCSS scales only for which complete data were available for all participants. For the purposes of this part of the analysis, we included all 47 patients meeting criteria for “definite” or “probable” CIDP, including the eight IVIg-non-responders. This was done because the results from the previous trial included both IVIg-responders and non-responders [13]. The corresponding modified-INCAT scores and improvement levels were directly obtained from the ONLS scores for each one of our 47 treated subjects [7]. Modified-INCAT improvement levels were similar to those of the IVIg arm of the ICE Study [13], [mean: 1.45 (SD: 1.73) vs. mean: 1.2 (SD: 1.5); *p* = 0.47]. MRCSS improvement was greater in our 47 patients as compared to the IVIg arm of the ICE Study [13] [mean: 10.15 (SD: 8.0) vs. mean: 4.4 (SD: 6.5); *p* < 0.001].

Initial courses had been administered in our patients using DW, over 5 days, although 12 patients (30.8%) had received higher doses based on recorded weight at first treatment outside our institution. Mean initial IVIg dose at 2 g/kg, using DW, was 152.8 g per course. Mean initial theoretical IVIg dose, using recorded weight at 2 g/kg, was 177.4 g per course. This implied administration of a mean extra 24.6 g/patient/course in initial disease stages as per our treatment protocol.

Mean final IVIg infusion duration in the 29 patients remaining on long-term treatment was 2.79 days (SD: 1.15) and mean final IVIg infusion frequency was every 4.34 weeks (SD: 1.70). Mean final IVIg dose infused was of 22.06 g/patient/week (SD: 15.29). However, 37.9% of patients required ≤ 15 g/week, 62.1% required ≤ 20 g/week and 75.9% required ≤ 25 g/week. On the other hand, 24.1% required ≥ 30 g/week and 10.3% required ≥ 35 g/week. Thus, a significantly greater proportion of patients on on-going treatment had low IVIg dose requirements of ≤ 15 g/week, compared to high dose requirements of ≥ 35 g/week (11/29 vs. 3/29; *p* = 0.03).

Our main results for doses and costs as well as the hypothetical values with standard dosing, are summarised in Table 2. The current cost of IVIg was estimated at GBP 32.83/g (€ 37.75), this representing the average price of the three main brands used at our institution (July 2018). The mean IVIg drug cost was, therefore, of GBP 37,660/patient/year (€ 43,309) for those on on-going treatment. In view of the mean infusion frequency and duration and daily cost on our Day Case Unit (GBP 512; € 589), hospital infusion-related non-drug costs amounted to a mean of GBP 17,115/patient/year (€ 19,682). Total IVIg costs consequently amounted to a mean of GBP 54,775/patient/year (€ 62,991) for patients on on-going IVIg, with IVIg drug costs representing 68.8% of total IVIg-related costs.

**Table 2:** Mean costs of IVIg treatment in 29 patients with CIDP on long-term treatment: real costs using an outcome-measured treatment protocol vs. hypothetical costs using standard dosing at 1 g/kg every 3 weeks using (1) dosing weight and (2) recorded weight

	Mean Weekly IVIg Dose Used (g/week)	Mean Yearly IVIg Dose Used (g/year)	Mean Infusion Frequency (interval in weeks)	Mean Infusion Duration (days)	Mean Yearly IVIg Drug Costs GBP/patient (€)	Mean Yearly IVIg infusion related non-drug costs GBP/patient (€)	Mean Total Yearly IVIg related Costs GBP/patient (€)
Real IVIg consumption and costs incurred with Protocol and outcome measures used	22.06	1149	4.34	2.79	37,660 (€ 43,309)	17,115 (€ 19,682)	54,775 (€ 62,991)
Consumption and costs with hypothetical standard dosing at 1 g/kg/ 3 weeks using dosing weight	25.50	1326	3	2	43,532 (€ 50,062)	17,749 (€ 20,411)	61,281 (€ 70,473)
Consumption and costs with hypothetical standard dosing at 1 g/kg/ 3 weeks using recorded weight	29.60	1539	3	2	50,532 (€ 58,119)	17,749 (€ 20,411)	68,281 (€ 78,523)

Ten of the 39 patients (25.6%), were successfully taken off IVIg therapy during the study period as a result of disease remission. Age (*p* = 0.75) and gender (*p* = 1) distribution were similar in these ten patients as in the 29 remaining on treatment.

Details relating to cost savings achieved with our protocol are provided in Table 3. Applying standard dosing treatment regimens of 1 g/kg administered every 3 weeks using DW, mean total IVIg costs were higher than the actual expenses in our cohort, by GBP 6,506/patient/year (€ 7,482), i.e., 11.9%. Applying standard dosing with recorded weight instead of DW, the mean total overspend, would have been of GBP 13,506/patient/year (€ 15,532), i.e., 24.7%.

**Table 3:** Mean savings achieved during IVIg treatment in 29 patients with CIDP on long-term treatment using an outcome-measured treatment protocol with cut-offs defining response vs. hypothetical costs using standard dosing at 1 g/kg every 3 weeks using dosing weight and recorded weight

	Mean IVIg dose saved in g/ patient/week	Mean IVIg dose saved in g/ patient/year	Rounded Number of avoided infusions sessions/year	Rounded Number of avoided Infusion days /year	Mean IVIg Drug Costs saved/patient/year GBP (€)	Mean IVIg non-drug Costs saved/ patient /year GBP (€)	Mean Total IVIg Costs saved/patient/year GBP (€)
Savings achieved compared to Standard Dosing at 1 g/kg/3 weeks using dosing weight	3.44	179	6	1	5872 (€ 6,753)	634 (€ 729)	6506 (€ 7,482)
Savings achieved compared to Standard Dosing of 1 g/kg/ 3 weeks using recorded weight	7.54	392	6	1	12,872 (€ 14,803)	634 (€ 729)	13,506 (€ 15,532)

## Discussion

The clinical and economic aspects of IVIg treatment for CIDP are complex and have not been studied specifically. Although central, the questions surrounding diagnostic accuracy, responder rate, minimum effective dose and frequency requirements, weight-based dosing, use of disability scales, implementation of functional score cut-off levels and remission rate, have not been included in analyses performed to date.

We here investigated the objective functional gain and costs of IVIg treatment in a UK cohort of patients with CIDP attending our unit, all fulfilling EFNS/PNS criteria for a “definite” or “probable” diagnosis, monitored using validated disability scales with defined cut-offs for clinical benefit and implementing a dose-altering protocol. The resulting analysis is, to our knowledge, the first to consider all these points and, therefore, provides accurate results directly in relation to quantified clinical benefit and real-life costs of IVIg-treated CIDP. Clinical and economic data were compared with those of existing standard dosing regimens. To our knowledge, this is the first study to compare these aspects with different IVIg treatment strategies for CIDP.

We found that mean IVIg drug costs amounted to GBP 37,660/patient/year (€ 43,309), for subjects on on-going treatment. These appear higher than previously reported in the UK, which were of GBP 34,215 (€ 39,347) for total IVIg costs [4]. Considering identical proportions to our findings of drug and non-drug costs for that study conclude to a figure of GBP 23,523 (€ 27,051) for IVIg drug costs only. As that analysis described a mean IVIg dose per course of 135 g and quoted estimated costs of GBP 33/g (€ 37.95), this indicates that patients received a mean of 713 g/ year (13.71 g/week) and a mean of 5.28 courses yearly. This equates to a mean infusion frequency of every 9.85 weeks, i.e., over twice less frequently than what is reported as average needs in relapsing CIDP, i.e., about every 4 weeks, as indicated from different cohorts [11, 15, 16]. It is consequently possible that, in this previously studied cohort, for which clinical amelioration levels were not described, undertreatment may have occurred, explaining lower costs.

The mean IVIg dose administered in our long-term patients with CIDP was of 22.06 g/week. This compares cost-wise favourably to the dose of 1 g/kg/3 weeks, found effective in stabilising the condition in the medium and long-term in recent trials [13, 14]. It is noteworthy that marked inter-subject dose requirement variations were observed, as we and others had previously reported [3, 15, 16]. The majority (62.1%) of our patients had requirements of ≤ 20 g/week. Concurrently, our protocol allowed at higher dose and cost, stabilising nearly a quarter of long-term IVIg-responding subjects with requirements of ≥ 30 g/week, at optimal levels of neurological function. Very high IVIg needs are described in a minority of patients with CIDP [17] and finding and implementing the right dose in these subjects may be challenging, particularly in view of existing guidelines and monitoring by local hospital-based regulating bodies. This is, however, imperative to ensure optimum patient benefit and likely to otherwise also favourably impact on primary and social care costs, which were not considered here.

From the clinical perspective, the modified-INCAT score improvement in our cohort was similar to that reported in the ICE Study, considering a similar-sized IVIg-treated group, using a standard dosing regimen [13]. The MRCSS improvement was otherwise significantly greater in our patients. These findings may partly relate to a greater proportion of our patients being severely weak at baseline and improving to greater levels of muscle strength, although to similar levels of function. The results indicate non-inferiority of the clinical benefit achieved with our protocol in comparison to standard dosing at 1 g/kg/3 weeks.

We and others have previously described the absence of correlation of recorded weight with IVIg dose requirements in CIDP [3, 15, 16]. The current analysis is, however, the first, to our knowledge, to demonstrate the clinical equivalence of DW to recorded weight in the initial stages of CIDP treatment. This is important as the use of recorded weight remains widespread both in clinical practice and research. Use of DW allowed mean savings of GBP 808/patient/course (€ 929) in early treatment stages as per our protocol. Furthermore, administration of lower IVIg doses may be protective against the risk of thromboembolic complications [18].

Our IVIg-responder rate, whether considering the above-described definition used in our clinical practice (39/47; 83%), or modified-INCAT score amelioration only (33/47; 70.2%), as well as our remission rate (25.6%), were similar to those previously reported [14, 19, 20]. This suggests that our cohort is comparable to previously described CIDP populations and that our findings have wide applicability.

Our analysis is limited by its retrospective design and consideration of treatment costs only within the hospital environment. The comparative analysis with historical controls from the ICE Study is a drawback although use of a contemporary control group was not possible in view of the design of this cohort study of clinical practice within our unit. The final documented dose analysis we used for cost calculations is subject to possible future change in treatment requirements in some patients. Nevertheless, the extended retrospective review over a 4-year period, offers a global and protracted view of IVIg requirements in a CIDP cohort. It is finally possible that the IVIg doses used were high in a relatively large number of our patients. A proportion of subjects were referred to our tertiary centre as a result of refractoriness and high-dose and/or high-frequency IVIg therapy is known to be effective in some patients in this setting [21].

Subcutaneous immunoglobulin (SCIg) therapy may represent the future of CIDP treatment [22]. However, drug costs will remain a predominant question, representing in our analysis, nearly 70% of immunoglobulin treatment costs. Therefore, similarly protocolised SCIg treatment may require consideration in comparison to standard dosing in future.

In conclusion, total IVIg costs are for CIDP, without compromising on clinical benefit, 10–25% lower with an individualised treatment protocol, than with standard dosing regimens. Our cost variation estimates, using DW and recorded weight from a real patient cohort, indicate that use of DW should be implemented at treatment initiation to avoid unnecessary excessive IVIg expenditure in the first treatment stages. More importantly for the long-term, use of a subsequently weight-independent, outcome-measured, dose-modifying protocol with defined cut-offs for clinical benefit, appears optimal for management of both clinical and economic aspects of IVIg-treated patients with CIDP.
